# Supplementary material for: Cytotoxic Marine Alkaloid 3,10-Dibromofascaplysin Induces Apoptosis and Synergizes with Cytarabine Resulting in Leukemia Cell Death
Source: Mar Drugs. 2021 Aug 27;19(9):489. doi: 10.3390/md19090489 (PMC8468638; doi:10.3390/md19090489)

Figure S1. ^{1}H NMR spectra of fascaplysin (Fas)

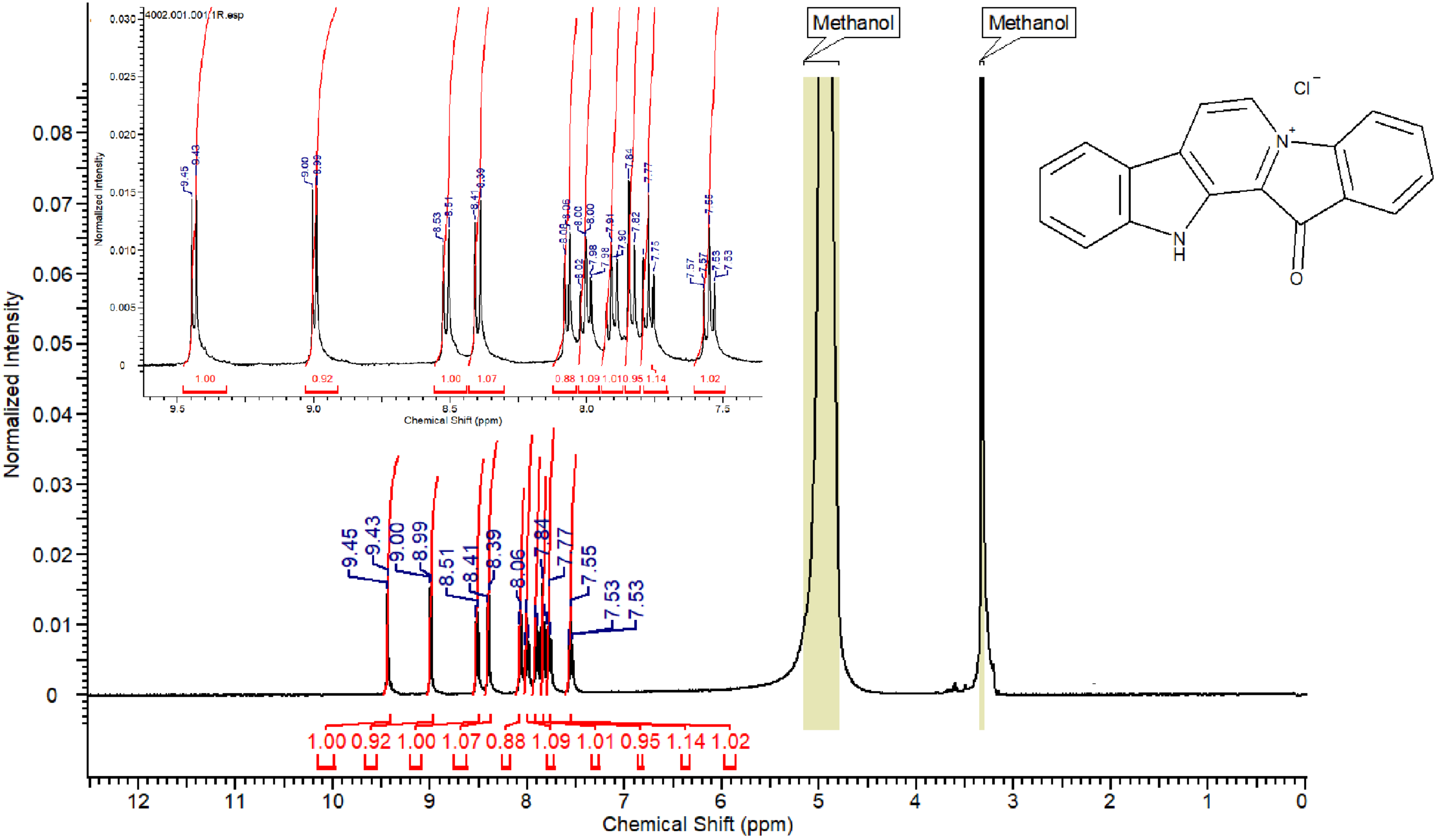

Figure S2. ^{1}H NMR spectra of 3.10-dibromofascaplysin (DBF)

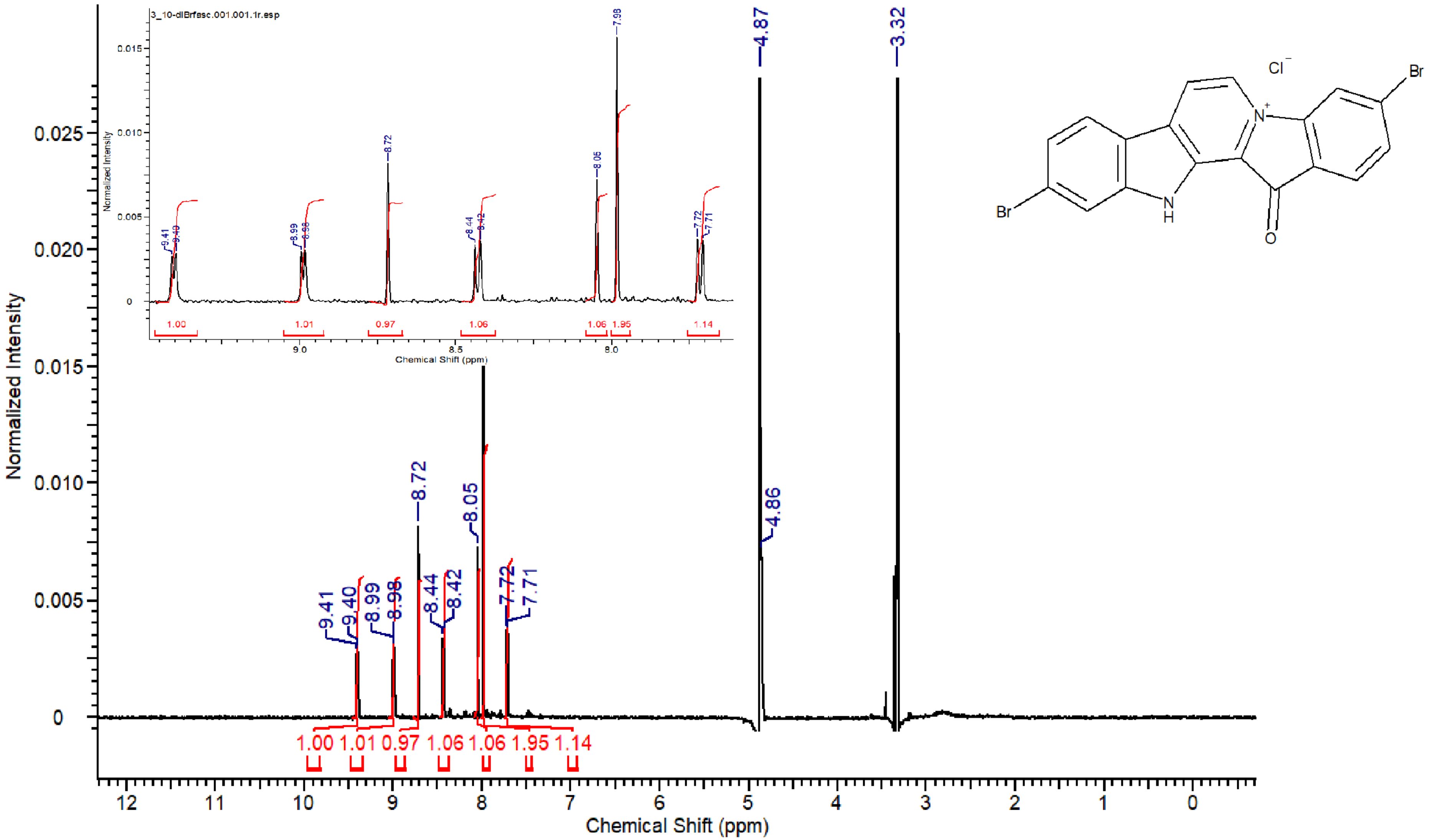

Figure S3. ^{1}H NMR spectra of 3-bromofascaplysin (3BF)

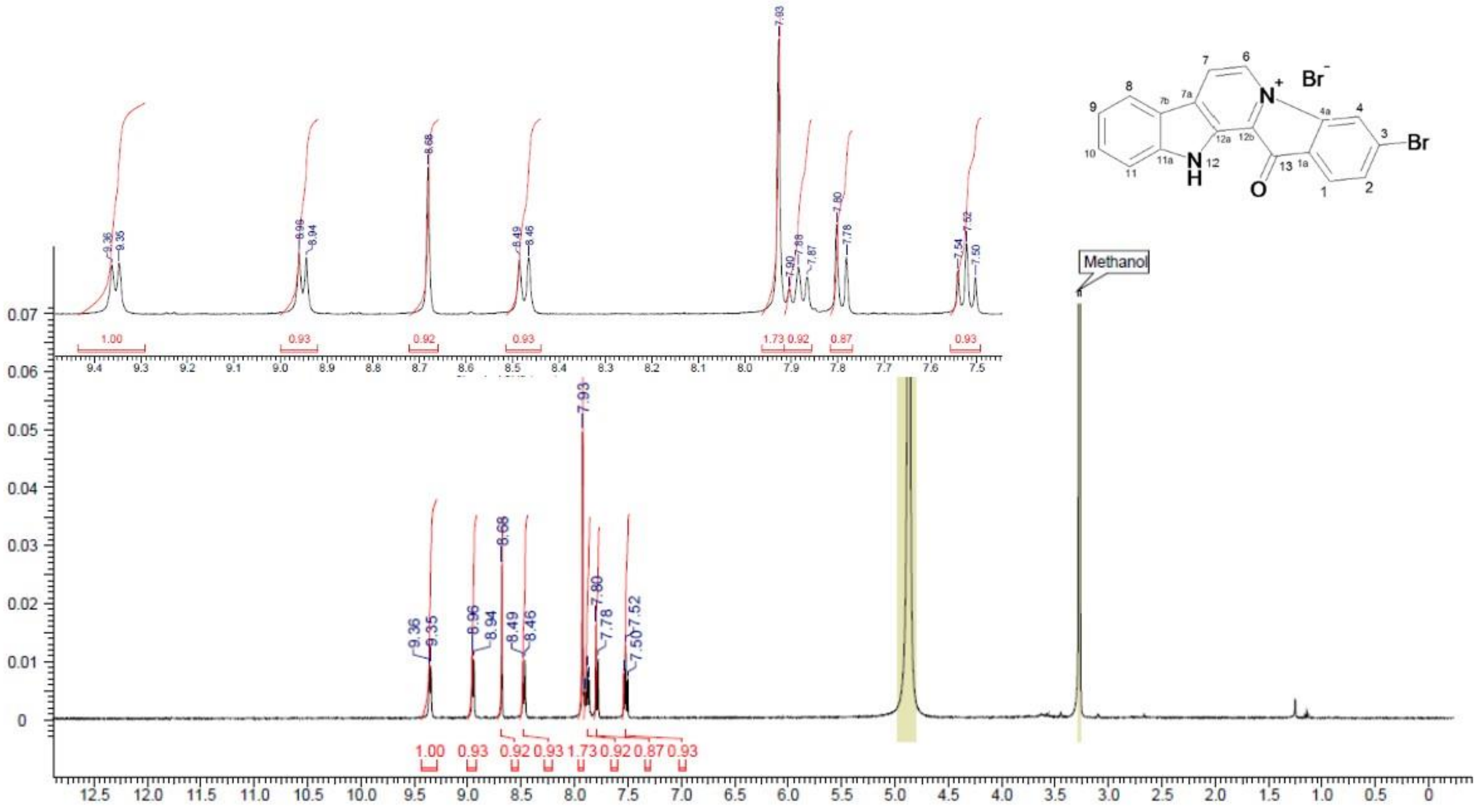

Figure S4. ^{1}H NMR spectra of 2-bromofascaplysin (2BF)

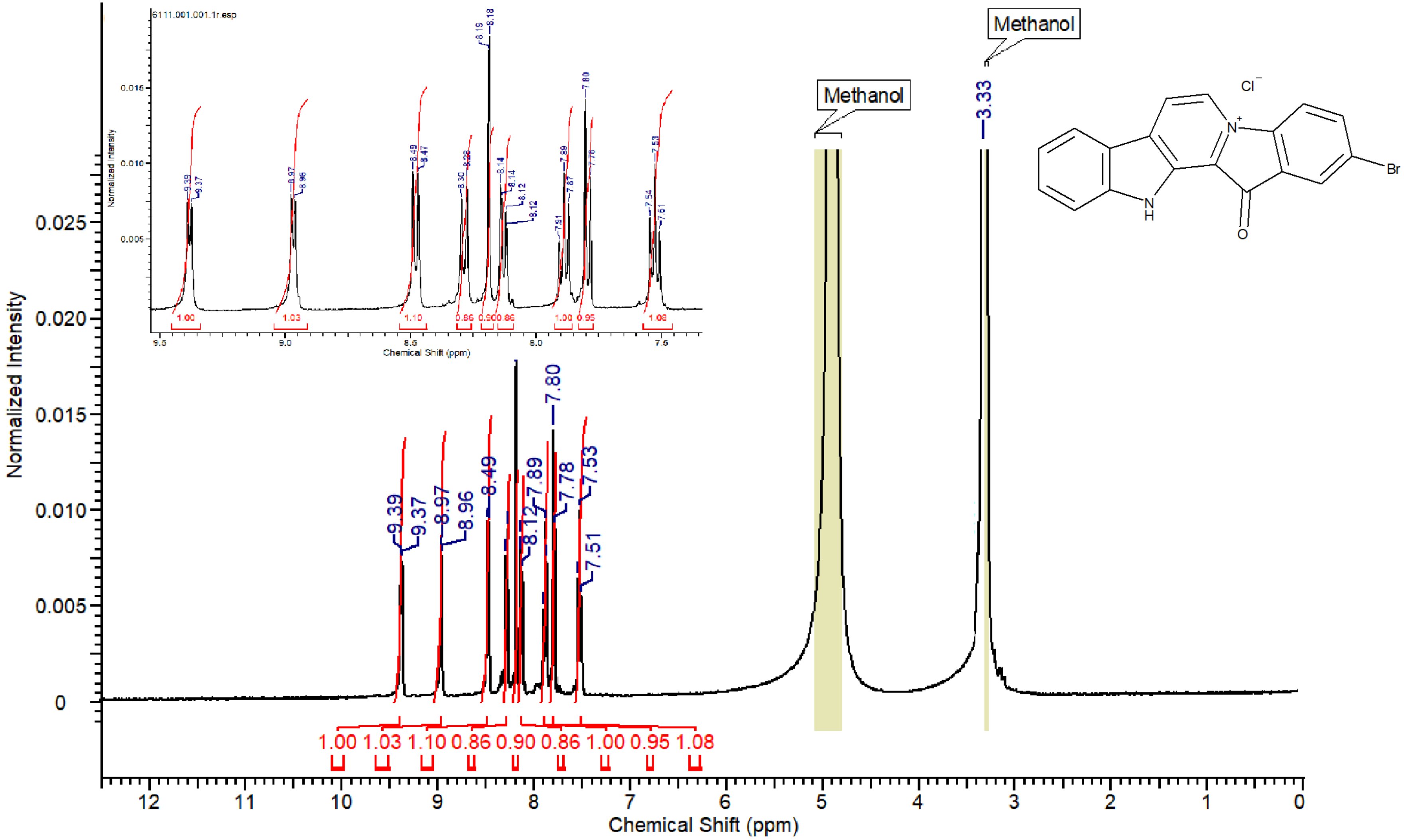

Figure S5. ^{1}H NMR spectra of 10-bromofascaplysin (10BF)

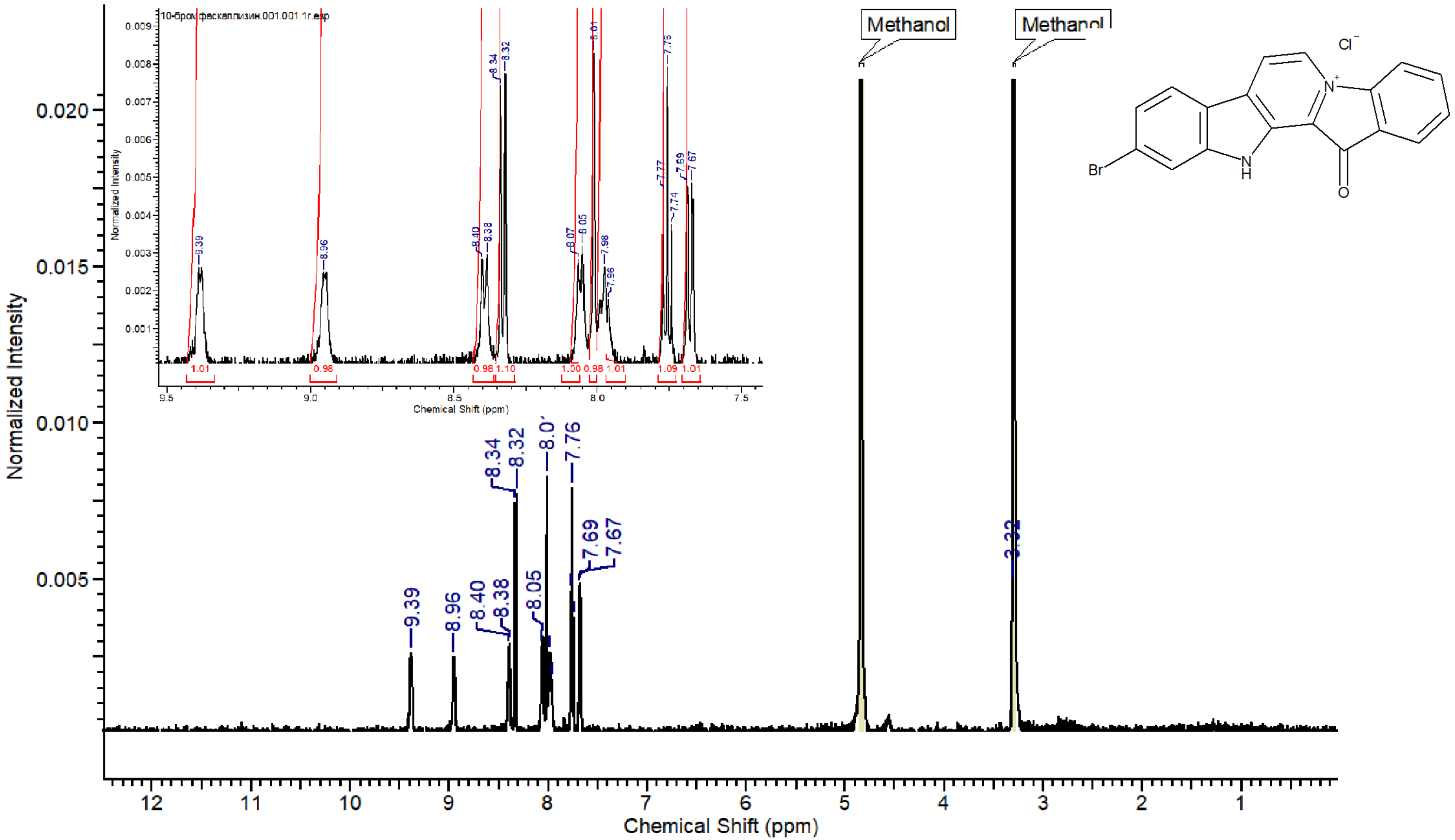

Supplement: Supplementary file 1 [file marinedrugs-19-00489-s001.zip › Figures S1-S5.pdf]
