# Supplementary material for: Cytotoxic Marine Alkaloid 3,10-Dibromofascaplysin Induces Apoptosis and Synergizes with Cytarabine Resulting in Leukemia Cell Death
Source: Mar Drugs. 2021 Aug 27;19(9):489. doi: 10.3390/md19090489 (PMC8468638; doi:10.3390/md19090489)
Supplement: Supplementary file 1 [file marinedrugs-19-00489-s001.zip › Table S1.pdf]

Supplementary table 1. List of primers

	PRIMER 1	PRIMER 2
ABL1	CCTGTCCCAGGTGTATGAGC	GCTCCTTTTCCACTTCGTCTGA
BCL2	TGAACTGGGGGAGGATTGTG	CGTACAGTTCCACAAAGGCA
BCL2L1	GCGTGGAAAGCGTAGACAAG	GTCAGGAACCAGCGGTTGAA
CCNA1	GCCTCCTGTCTGGTGGGA	TGCAGTGCATTGCTTCAGAC
CCNB1	CCTCTCCAAGCCCAATGGAA	TGGTCTGACTGCTTGCTCTT
CCND1	TGTCCTACTACCGCCTCACACGCTTCCTCTCCG	TCCTCTTCCTCCTCCTCGGCGGCCTTG
CCND2	GTTCCTGGCCTCCAAACTCA	CTTGATGGAGTTGTCGGTGTAAAT
CCNE1	GGGAGCGGGATGCGA	GGGTCTGCACAGACTGCATTA
CDKN1B	GGCTAACTCTGAGGACACGC	TGGGGAACCGTCTGAAACAT
CTNNB1	AAAATGGCAGTGCGTTTAG	TTTGAAGGCAGTCTGTCGTA
E2F1	TGACCCAGGACCTCCGACAG	GCCTTGTTTGCTCTTAAGGGAGAT
FLT3	GTGCTTTGCGATTCACAGGG	GCACCTTATGTCCGTCCCAA
GAPDH	GAGCCCGCAGCCTCCCGCT	GCGCCCAATACGACCAAATC
GSK3B	GGAACTCCAACAAGGGAGCA	CCGCACTCCTGAGGTGAAAT
HOXA10	AGGTGGACGCTGCGGCTAATCTCTA	GCCCCTTCCGAGAGCAGCAAAG
KIT	CATCATGGAGGATGACGAGT	CGACCATGAGTAAGGAGGAT
MYC	TGAGGAGACACCGCCCAC	CAACATCGATTTCTTCCTCATCTTC
NFKB	ACCCGGCTTCAGAATGGCA	GGTATGGGCCATCTGCTGTT
PDGFRB	CACCGCAACAAACACACCTT	CAGCATGGGCACATAGTCCA
TP53	ACCTATGGAAACTACTTCCTGAAA	CTGGCATTCTGGGAGCTTCA
VEGFR2	AGACCGGCTGAAGCTAGGTA	CGATGCTCACTGTGTGTTGC
